# Supplementary material for: Pan-cancer study detects genetic risk variants and shared genetic basis in two large cohorts
Source: Nat Commun. 2020 Sep 4;11:4423. doi: 10.1038/s41467-020-18246-6 (PMC7473862; doi:10.1038/s41467-020-18246-6)
Supplement: Supplementary file 1 — Supplementary Information [file 41467_2020_18246_MOESM1_ESM.pdf]

Pan-Cancer Study Detects Genetic Risk Variants and Shared Genetic Basis in Two Large Cohorts

Rashkin et al.

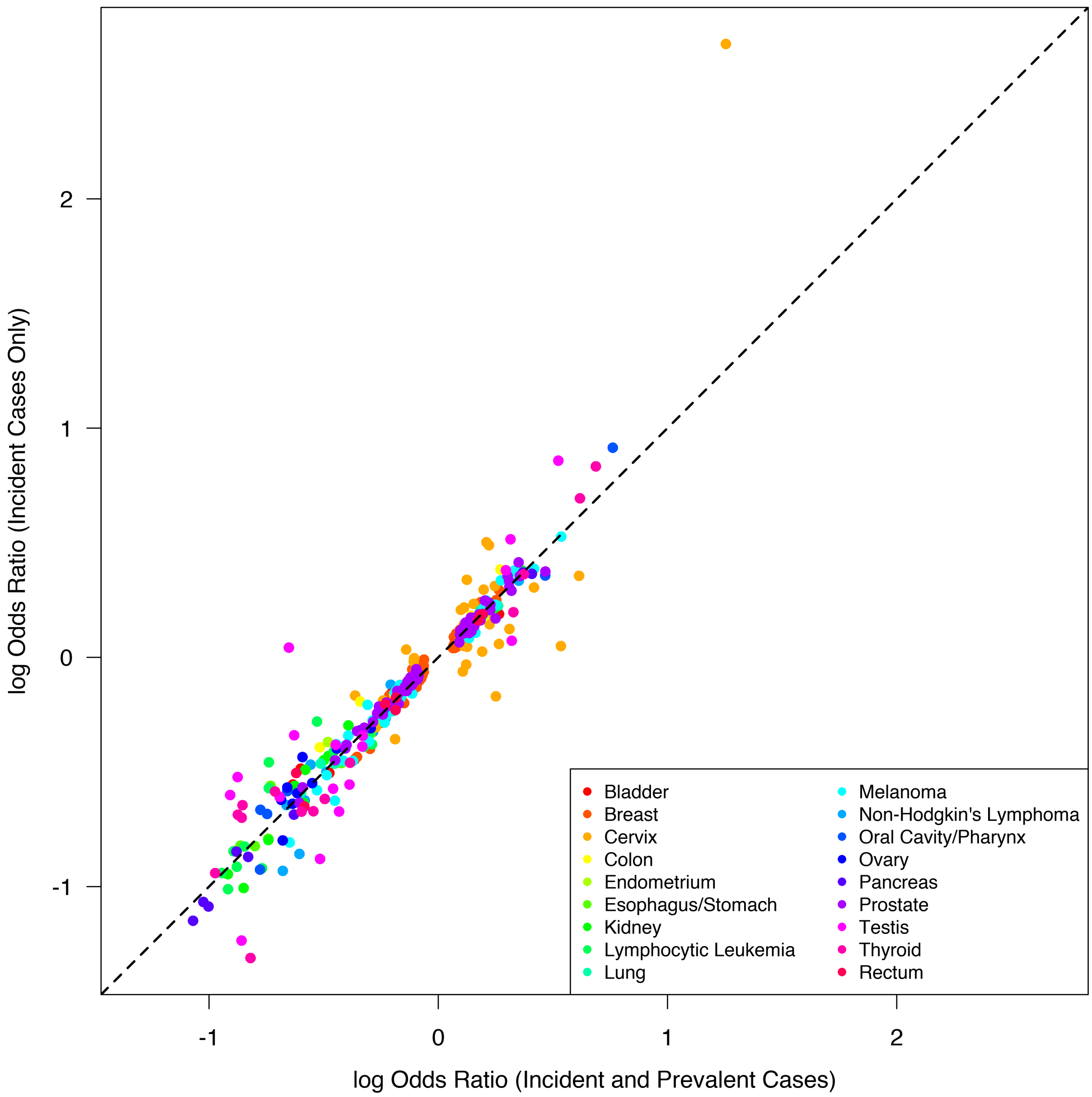

Supplementary Figure 1. Comparison of effect sizes (log odds ratios) for associations between variants and incident cancers only versus those obtained using both incident and prevalent cases. For all cancers, we compared associations for independent SNPs with $P<1x10^{-6}$ in the analysis with incident and prevalent cancers in UKB (2-95 SNPs per cancer). The effect estimates did not exhibit heterogeneity ($P > 0.05$/[number of SNPs per cancer]) and were highly correlated ($r^2=0.95$ across all 396 SNPs tested).

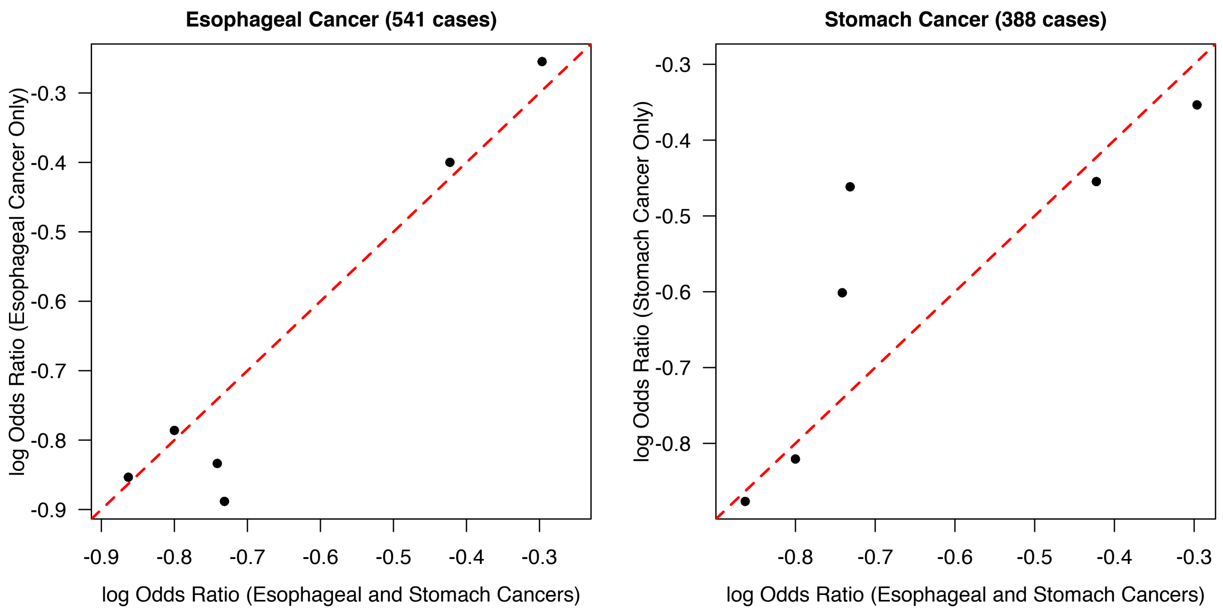

Supplementary Figure 2. **Comparison of effect sizes (log odds ratios) for associations between variants and esophageal (left) and stomach (right) cancers versus those obtained using both cancers.** We compared associations for independent SNPs with $P<1\text{x}10^{-6}$ in the analysis of the combined cancer phenotype in UKB alone (6 SNPs). The effect estimates did not exhibit heterogeneity (P > 0.05/6) and were highly correlated (r^2=0.98 comparing esophageal to combined and r^2=0.83 comparing stomach to combined).

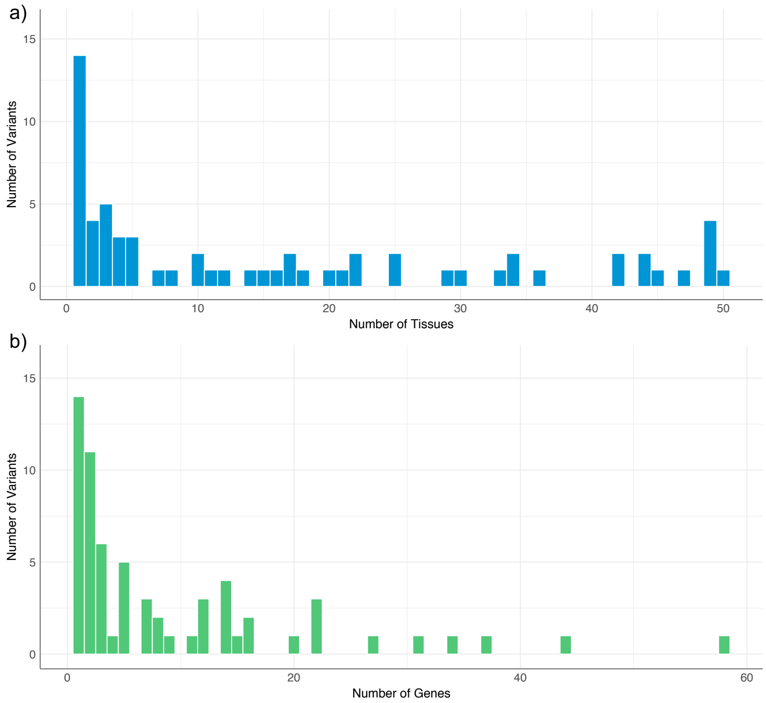

c)

Tissue	Genes	(%)
Whole Blood (BIOS QTL)	265	44.5%
Adipose	88	14.8%
Whole Blood (GTEx)	28	4.7%
Esophagus (mucosa, muscularis, junction)	28	4.7%
Artery (tibial, coronary, aorta)	27	4.5%
Skin (sun exposed, sun unexposed)	24	4.0%
Brain (11 structures)	22	3.7%
Thyroid	19	3.2%
Nerve (tibial)	15	2.5%
Muscle (skeletal)	14	2.3%
Cells: Cultured fibroblasts	13	2.2%
Testis	10	1.7%
Lung	9	1.5%
Heart	7	1.2%
Adrenal Gland	6	1.0%
Liver	5	0.8%
Cells: EBV-transformed lymphocytes	3	0.5%
Pancreas	3	0.5%
Colon (sigmoid, transverse)	3	0.5%
Breast (mammary tissue)	2	0.3%
Pituitary	2	0.3%
Prostate	1	0.2%
Small Intestine (terminal ileum)	1	0.2%
Stomach	1	0.2%

Supplementary Figure 3. Overview of significant (FDR<0.05) effects on gene expression observed for pleiotropic variants in BIOS-QTL and GTEx v8 data sets. Histograms showing distribution of eQTL effects for (a) tissues and (b) genes across 64 pleiotropic variants with significant effects on gene expression. (c) Distribution of tissues for 596 variant-gene pairs.

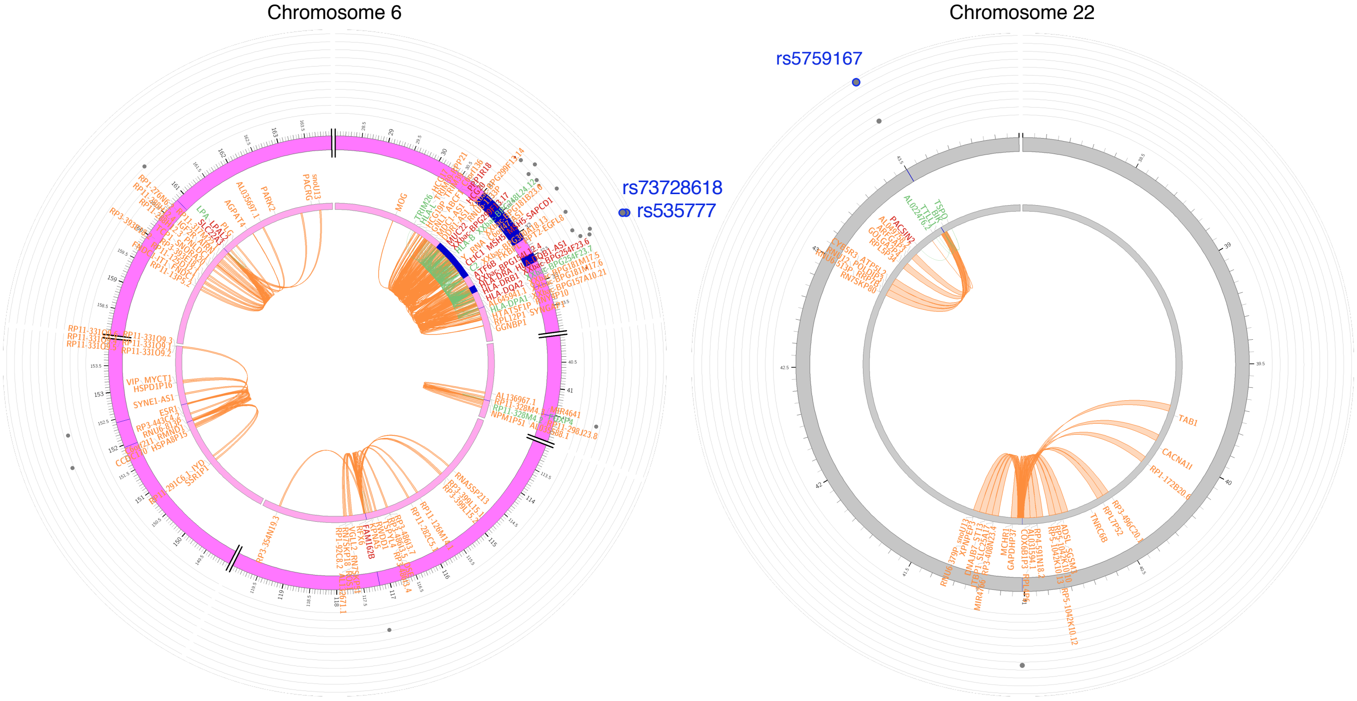

Supplementary Figure 4. Circos plots depicting three-dimensional chromatin interactions such as HiC loops and enhancer-promoter (EP) links (orange bands) and eQTL effects (green bands), for pleiotropic variants on chromosomes 6 and 22. If the gene is mapped only by chromatin interactions or only by eQTLs, it is colored orange or green, respectively. When the gene is mapped by both, it is colored red. All variants and links are plotted, but only variants with significant EP effects are annotated.

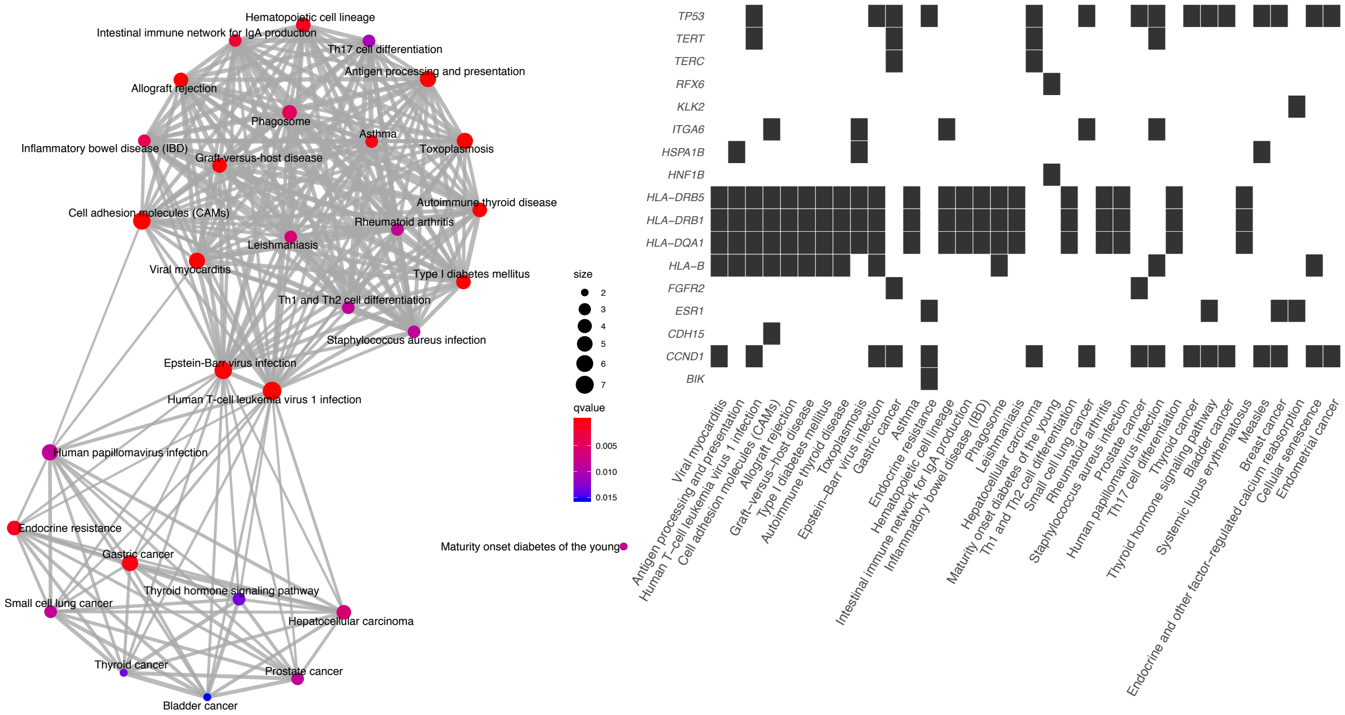

Supplementary Figure 5. Visualization of KEGG pathway analysis results for 100 pleiotropic ASSET variants. The enrichment map shows gene set networks with edges connecting overlapping sets. The top 30 significantly enriched (FDR *q*<0.05) gene sets are plotted. The heatmap shows the genes included in each significantly enriched gene set.

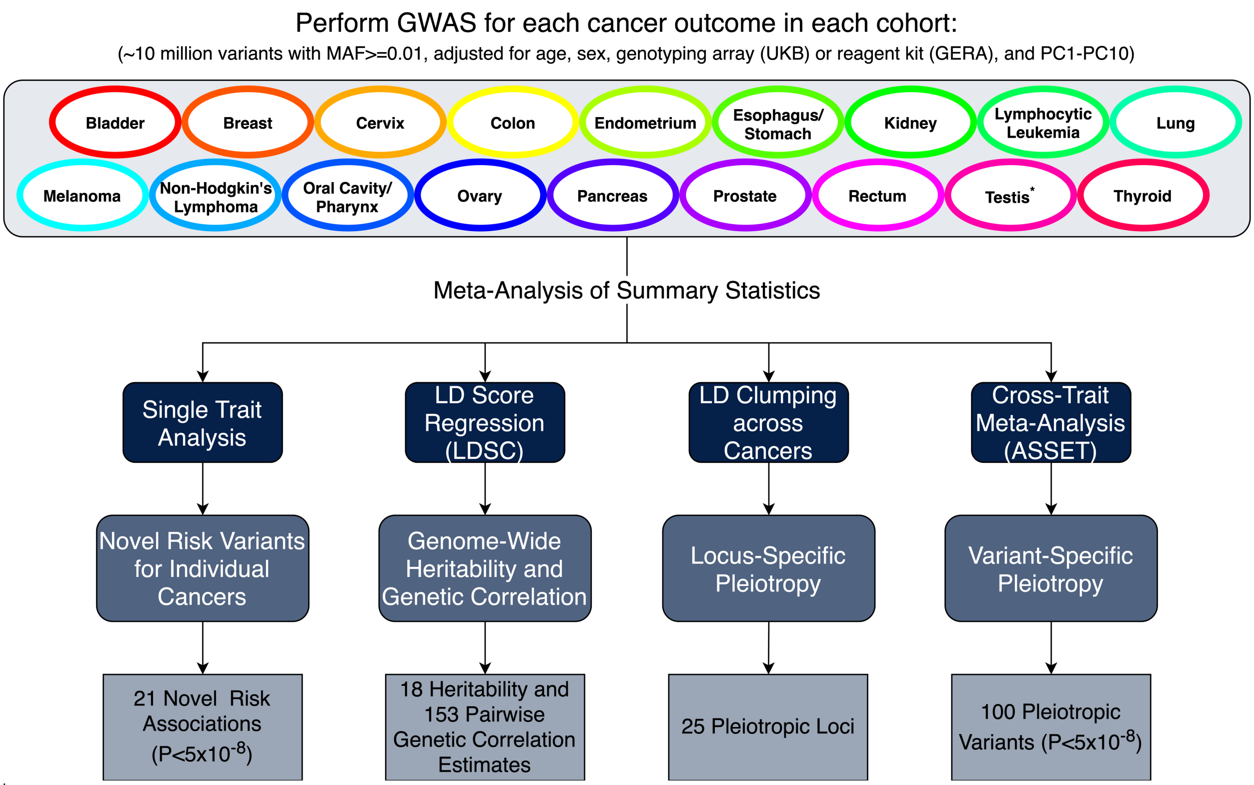

*Testicular cancer only analyzed in UKB

Supplementary Figure 6. Flowchart of overall approach used across analyses and major results from each. First, we undertook individual cancer genome-wide association studies (GWAS) in two cohorts (UK Biobank [UKB] and Kaiser Permanente Genetic Epidemiology Research on Adult Health and Aging [GERA]). Based on those results, we used four approaches to assess: 1) novel risk variants; 2) heritability and genetic correlation; 3) locus-specific pleiotropy; and 4) variant-specific pleiotropy.

Supplementary Table 1. Pleiotropic regions identified by LD clumping – regions are centered around index variant and additional variants were clumped if they were associated with any cancer with $P<1x10^{-6}$, were within 500kb of index, and had LD $r^2>0.5$ with index.

Index Variant	Chromosome	Position	Locus	Number of Additional Variants Clumped with Index	Cancer Sites Associated with SNPs in Region
rs2736109	5	1296759	5p15.33	1	Breast, Melanoma
rs31490	5	1344458	5p15.33	44	Cervix, Melanoma, Pancreas
rs9265490	6	31297343	6p21.33	1	Cervix, Non-Hodgkin's Lymphoma
rs9281573	6	31720525	6p21.33	1	Cervix, Non-Hodgkin's Lymphoma
6:32381852_GAA_G	6	32381852	6p21.32	8	Cervix, Non-Hodgkin's Lymphoma
rs9268805	6	32423399	6p21.32	3	Cervix, Non-Hodgkin's Lymphoma
rs3997868	6	32578590	6p21.32	9	Cervix, Non-Hodgkin's Lymphoma
rs4530903	6	32581889	6p21.32	382	Cervix, Non-Hodgkin's Lymphoma
rs11751024	6	32586236	6p21.32	4	Cervix, Non-Hodgkin's Lymphoma
6:32596798_AG_A	6	32596798	6p21.32	34	Cervix, Non-Hodgkin's Lymphoma
rs114786106	6	32596878	6p21.32	4	Cervix, Non-Hodgkin's Lymphoma
rs9272050	6	32599071	6p21.32	2	Cervix, Non-Hodgkin's Lymphoma
rs9272232	6	32602630	6p21.32	7	Cervix, Non-Hodgkin's Lymphoma
rs28383373	6	32607193	6p21.32	2	Cervix, Non-Hodgkin's Lymphoma
rs4713570	6	32626040	6p21.32	6	Cervix, Non-Hodgkin's Lymphoma
rs28414666	6	32626451	6p21.32	1	Cervix, Non-Hodgkin's Lymphoma
rs62516012	8	128335526	8q24.21	34	Breast, Prostate
rs7835046	8	128376926	8q24.21	2	Breast, Prostate
rs10505477	8	128407443	8q24.21	39	Colon, Prostate, Rectum
rs11985829	8	128409232	8q24.21	3	Colon, Prostate
rs6998254	8	128428795	8q24.21	3	Colon, Prostate
rs9297754	8	128429262	8q24.21	2	Colon, Prostate
rs7842552	8	128431694	8q24.21	1	Colon, Prostate
rs78378222	17	7571752	17p13.1	1	Melanoma, Lymphocytic Leukemia
rs6507874	18	46448805	18q21.1	1	Colon, Rectum

Supplementary Table 2. Significantly enriched KEGG pathways for the 100 one- and bidirectional pleiotropic variants from the ASSET analysis.

Pathway ID		Enrichment P	qFDR	Gene IDs
hsa05416	Viral myocarditis	1.15E-06	6.80E-05	HLA-DRB5/HLA-B/HLA-DRB1/HLA-DQA1/CCND1
hsa04612	Antigen processing and presentation	4.29E-06	7.01E-05	HLA-DRB5/HSPA1B/HLA-B/HLA-DRB1/HLA-DQA1
hsa05166	Human T-cell leukemia virus 1 infection	4.35E-06	7.01E-05	TERT/HLA-DRB5/HLA-B/HLA-DRB1/HLA-DQA1/CCND1/TP53
hsa04514	Cell adhesion molecules (CAMs)	5.83E-06	7.01E-05	CDH15/HLA-DRB5/HLA-B/HLA-DRB1/HLA-DQA1/ITGA6
hsa05330	Allograft rejection	5.95E-06	7.01E-05	HLA-DRB5/HLA-B/HLA-DRB1/HLA-DQA1
hsa05332	Graft-versus-host disease	8.11E-06	7.96E-05	HLA-DRB5/HLA-B/HLA-DRB1/HLA-DQA1
hsa04940	Type I diabetes mellitus	9.84E-06	8.28E-05	HLA-DRB5/HLA-B/HLA-DRB1/HLA-DQA1
hsa05320	Autoimmune thyroid disease	2.28E-05	1.66E-04	HLA-DRB5/HLA-B/HLA-DRB1/HLA-DQA1
hsa05145	Toxoplasmosis	2.53E-05	1.66E-04	HLA-DRB5/HSPA1B/HLA-DRB1/HLA-DQA1/ITGA6
hsa05169	Epstein-Barr virus infection	3.49E-05	2.05E-04	HLA-DRB5/HLA-B/HLA-DRB1/HLA-DQA1/CCND1/TP53
hsa05226	Gastric cancer	9.94E-05	5.33E-04	TERT/TERC/FGFR2/CCND1/TP53
hsa05310	Asthma	1.28E-04	6.29E-04	HLA-DRB5/HLA-DRB1/HLA-DQA1
hsa01522	Endocrine resistance	2.55E-04	1.12E-03	CCND1/BIK/TP53/ESR1
hsa04640	Hematopoietic cell lineage	2.65E-04	1.12E-03	HLA-DRB5/HLA-DRB1/HLA-DQA1/ITGA6
hsa04672	Intestinal immune network for IgA production	5.05E-04	1.98E-03	HLA-DRB5/HLA-DRB1/HLA-DQA1
hsa05321	Inflammatory bowel disease (IBD)	1.16E-03	4.26E-03	HLA-DRB5/HLA-DRB1/HLA-DQA1
hsa04145	Phagosome	1.34E-03	4.65E-03	HLA-DRB5/HLA-B/HLA-DRB1/HLA-DQA1
hsa05140	Leishmaniasis	1.89E-03	6.01E-03	HLA-DRB5/HLA-DRB1/HLA-DQA1
hsa05225	Hepatocellular carcinoma	1.94E-03	6.01E-03	TERT/TERC/CCND1/TP53
hsa04950	Maturity onset diabetes of the young	3.13E-03	8.29E-03	HNF1B/RFX6
hsa04658	Th1 and Th2 cell differentiation	3.14E-03	8.29E-03	HLA-DRB5/HLA-DRB1/HLA-DQA1
hsa05222	Small cell lung cancer	3.14E-03	8.29E-03	CCND1/ITGA6/TP53
hsa05323	Rheumatoid arthritis	3.24E-03	8.29E-03	HLA-DRB5/HLA-DRB1/HLA-DQA1
hsa05150	Staphylococcus aureus infection	3.54E-03	8.36E-03	HLA-DRB5/HLA-DRB1/HLA-DQA1
hsa05215	Prostate cancer	3.65E-03	8.36E-03	FGFR2/CCND1/TP53
hsa05165	Human papillomavirus infection	3.69E-03	8.36E-03	TERT/HLA-B/CCND1/ITGA6/TP53
hsa04659	Th17 cell differentiation	4.80E-03	0.010	HLA-DRB5/HLA-DRB1/HLA-DQA1
hsa05216	Thyroid cancer	6.28E-03	0.013	CCND1/TP53
hsa04919	Thyroid hormone signaling pathway	6.46E-03	0.013	CCND1/TP53/ESR1
hsa05219	Bladder cancer	7.67E-03	0.015	CCND1/TP53
hsa05322	Systemic lupus erythematosus	8.77E-03	0.017	HLA-DRB5/HLA-DRB1/HLA-DQA1
hsa05162	Measles	9.71E-03	0.018	HSPA1B/CCND1/TP53
hsa05224	Breast cancer	0.012	0.021	CCND1/TP53/ESR1
hsa04961	Endocrine and other factor-regulated calcium reabsorption	0.013	0.022	KLK2/ESR1
hsa04218	Cellular senescence	0.014	0.024	HLA-B/CCND1/TP53
hsa05213	Endometrial cancer	0.015	0.024	CCND1/TP53

Supplementary Table 3. Sample sizes for UK Biobank (UKB) and Kaiser Permanente Genetic Epidemiology Research on Adult Health and Aging (GERA).

	UKB		GERA	
Total Controls	359,825		50,525	
Female		189,855		29,801
Male		169,970		20,724
Total Cases	48,961		16,001	
Bladder		1,550		692
Breast		13,903		3,978
Cervix		5,998		565
Colon		2,897		896
Endometrium		1,414		623
Esophagus/Stomach		929		162
Kidney		1,021		317
Lymphocytic Leukemia		594		258
Lung		1,728		757
Melanoma		4,271		2,506
Non-Hodgkin's Lymphoma		1,760		640
Oral Cavity/Pharynx		930		293
Ovary		1,006		253
Pancreas		471		192
Prostate		7,441		3,351
Rectum		1,808		283
Testis		713		N/A
Thyroid		527		235

Supplementary Table 4. Case characteristics of UK Biobank (UKB) and Kaiser Permanente Genetic Epidemiology Research on Adult Health and Aging (GERA) cases by cancer (numbers do not add up where data are missing).

	Bladder		Breast		Cervix		Colon		Endometrium		Esophagus/ Stomach	
	UKB	GERA	UKB	GERA	UKB	GERA	UKB	GERA	UKB	GERA	UKB	GERA
Diagnosis Relative to Specimen Collection, n												
Prevalent	804	2813	9205	2813	5730	524	1287	527	811	393	257	44
Incident	746	321	4698	1165	268	41	1610	369	603	230	672	118
Median Age at Diagnosis, years (IQR)	62.2 (12.6)	71 (14)	55.1 (12.6)	62 (17)	37.0 (11.4)	38 (16)	62.2 (11.3)	71 (15)	59.4 (10.3)	64 (14.5)	64.5 (9.6)	74 (13.8)
Grade at Diagnosis, n												
1-2*		297		2327		22		634		426		52
3-4**		278		897		37		132		109		73
Stage at Diagnosis, n												
0-1†		624		3032		546		435		520		62
2-7††		54		912		17		449		95		92

	Kidney		Lung		Lympho-cytic Leukemia		Melanoma		Non-Hodgkin's Lymphoma		Oral Cavity/ Pharynx	
	UKB	GERA	UKB	GERA	UKB	GERA	UKB	GERA	UKB	GERA	UKB	GERA
Diagnosis Relative to Specimen Collection, n												
Prevalent	462	163	271	227	291	149	2578	1505	938	343	510	169
Incident	559	154	1457	530	303	104	1693	1001	822	294	420	123
Median Age at Diagnosis, years (IQR)	61.3 (12.1)	69 (13)	65.8 (9.1)	73 (14)	62.0 (11.5)	71 (16)	56.6 (16.4)	65 (18)	59.6 (14.7)	68 (16)	57.5 (12.5)	66 (16)
Grade at Diagnosis, n												
1-2*		129		221		0		15		7		149
3-4**		101		162		0		5		7		70
Stage at Diagnosis, n												
0-1†		236		230		0		2407		211		175
2-7††		79		522		258		73		394		111

	Ovary		Pancreas		Prostate		Rectum		Testis		Thyroid	
	UKB	GERA	UKB	GERA	UKB	GERA	UKB	GERA	UKB	GERA	UKB	GERA
Diagnosis Relative to Specimen Collection, n												
Prevalent	595	156	51	21	3028	2465	939	205	664		370	159
Incident	411	97	420	171	4413	885	869	78	49		157	76
Median Age at Diagnosis, years (IQR)	56.6 (16.0)	62 (20)	65.9 (8.9)	76 (12)	64.5 (7.8)	67 (10)	60.7 (11.5)	64 (14.5)	40.2 (14.4)		51.9 (18.2)	56 (19)
Grade at Diagnosis, n												
1-2*		47		37		2284		190				28
3-4**		104		21		923		22				5
Stage at Diagnosis, n												
0-1†		86		30		2832		185				168
2-7††		161		156		397		89				62

* Well or moderately differentiated
** Poorly or undifferentiated
† In situ or localized
†† Regional or distant metastases

Supplementary Table 5. Lifetime risk of each cancer based on SEER 2012-2014.

Cancer Site	Lifetime Risk
Bladder	0.0257
Breast	0.1495
Cervix*	0.0060
Colon**	0.0304
Endometrium	0.0294
Esophagus/Stomach	0.0125
Kidney	0.0169
Lung	0.0650
Lymphocytic Leukemia	0.0075
Melanoma	0.0436
Non-Hodgkin's Lymphoma	0.0221
Oral Cavity/Pharynx	0.0119
Ovary***	0.0133
Pancreas	0.0155
Prostate	0.1082
Rectum**	0.0132
Testis	0.0047
Thyroid	0.0127

* SEER 2012-2014 only reports lifetime risk of invasive cervical cancer; our data include invasive and *in situ*.
** SEER 2012-2014 only reports lifetime risk of colorectal cancer (0.0436). From the American Cancer Society (https://www.cancer.org/cancer/colon-rectal-cancer/about/key-statistics.html, accessed April 15, 2019), colorectal cancer incidence can be partitioned into colon (69.66% of cases) and rectal (30.34%). These percentages were used to decompose the lifetime risk of colorectal cancer into lifetime risks of colon and rectal cancer.
*** SEER 2012-2014 only reports lifetime risk of invasive ovarian cancer, excluding borderline cases; our data include invasive and borderline.
