# Supplementary material for: Pan-cancer study detects genetic risk variants and shared genetic basis in two large cohorts
Source: Nat Commun. 2020 Sep 4;11:4423. doi: 10.1038/s41467-020-18246-6 (PMC7473862; doi:10.1038/s41467-020-18246-6)
Supplement: Supplementary file 2 — Descriptions of Additional Supplementary Files [file 41467_2020_18246_MOESM2_ESM.pdf]

Descriptions of Additional Supplementary Files

Supplementary Data 1. Previously Unreported variant associations with cancers from GWAS of UK Biobank (UKB) and Kaiser Permanente Genetic Epidemiology Research on Adult Health and Aging (GERA) cohorts.

Supplementary Data 2. Known GWAS variants detected at $P<1x10^{-6}$ in union set of all UK Biobank (UKB) and Kaiser Permanente Genetic Epidemiology Research on Adult Health and Aging (GERA) SNPs for each cancer that confirm previously reported risk variants for that cancer.

Supplementary Data 3. Cross-cancer genetic correlations and 95% confidence intervals (CIs) calculated via LD-score regression (LDSC) for all 153 cancer pairs.

Supplementary Data 4. One-directional pleiotropic variants with overall $P<1x10^{-6}$ and cancer sites with which they are associated.

Supplementary Data 5. Bidirectional pleiotropic variants with overall $P<1x10^{-6}$ and both directional $P<0.05$ and cancer sites with which they are associated.

Supplementary Data 6. Number of pleiotropic variants (of the 100 one- and bidirectional variants with overall pleiotropic $P<5x10^{-8}$) associated with each pair of cancers by type of pleiotropic effect.

Supplementary Data 7. Functional annotations for the 100 one- and bidirectional ASSET pleiotropic variants.

Supplementary Data 8. Cross-cancer genetic correlations and 95% confidence intervals (CIs) calculated via LD-score regression (LDSC) for all 153 cancer pairs calculated from summary statistics for genome-wide association analyses conducted in the UK Biobank alone.
