# Supplementary material for: Pan-cancer study detects genetic risk variants and shared genetic basis in two large cohorts
Source: Nat Commun. 2020 Sep 4;11:4423. doi: 10.1038/s41467-020-18246-6 (PMC7473862; doi:10.1038/s41467-020-18246-6)
Supplement: Supplementary file 11 — Reporting Summary [file 41467_2020_18246_MOESM11_ESM.pdf]

nature research

Corresponding author(s): John S. Witte and Lori C. Sakoda

Last updated by author(s): Jul 24, 2020

Reporting Summary

Nature Research wishes to improve the reproducibility of the work that we publish. This form provides structure for consistency and transparency in reporting. For further information on Nature Research policies, see Authors & Referees and the Editorial Policy Checklist .

Statistics

For all statistical analyses, confirm that the following items are present in the figure legend, table legend, main text, or Methods section.

n/a	Confirmed	
☐	☒	The exact sample size (*n*) for each experimental group/condition, given as a discrete number and unit of measurement
☐	☒	A statement on whether measurements were taken from distinct samples or whether the same sample was measured repeatedly
☐	☒	The statistical test(s) used AND whether they are one- or two-sided<br>*Only common tests should be described solely by name; describe more complex techniques in the Methods section.*
☐	☒	A description of all covariates tested
☐	☒	A description of any assumptions or corrections, such as tests of normality and adjustment for multiple comparisons
☐	☒	A full description of the statistical parameters including central tendency (e.g. means) or other basic estimates (e.g. regression coefficient) AND variation (e.g. standard deviation) or associated estimates of uncertainty (e.g. confidence intervals)
☐	☒	For null hypothesis testing, the test statistic (e.g. *F*, *t*, *r*) with confidence intervals, effect sizes, degrees of freedom and *P* value noted<br>*Give P values as exact values whenever suitable.*
☒	☐	For Bayesian analysis, information on the choice of priors and Markov chain Monte Carlo settings
☒	☐	For hierarchical and complex designs, identification of the appropriate level for tests and full reporting of outcomes
☐	☒	Estimates of effect sizes (e.g. Cohen's *d*, Pearson's *r*), indicating how they were calculated

Our web collection on statistics for biologists contains articles on many of the points above.

Software and code

Policy information about availability of computer code

Data collection	No software was used
Data analysis	R versions 3.2.0: https://cran.r-project.org<br>R package ASSET version 1.8.0: https://bioconductor.org/packages/release/bioc/html/ASSET.html<br>PLINK version 1.9 and 2.0: https://www.cog-genomics.org/plink/1.9/ and https://www.cog-genomics.org/plink/2.0/<br>Phenoscanner: http://www.phenoscanner.medschl.cam.ac.uk<br>LDlink: https://ldlink.nci.nih.gov/?tab=home<br>KING version 2.0: http://people.virginia.edu/~wc9c/KING/<br>GCTA version 1.92.2beta: https://cnsgenomics.com/software/gcta/#Overview<br>LDSC version 1.0.0: https://github.com/bulik/ldsc/<br>FUMA version 1.3.5: https://fuma.ctglab.nl/<br>DeepSEA: http://deepsea.princeton.edu/job/analysis/create/<br>CADD version 1.4: https://cadd.gs.washington.edu/<br>R packages ReactomePA and clusterProfiler for pathway analysis: https://bioconductor.org/packages/release/bioc/html/ReactomePA.html and https://bioconductor.org/packages/release/bioc/html/clusterProfiler.html<br>ImmuneSigDB (C7): http://software.broadinstitute.org/gsea/msigdb/collections.jsp<br>EIGENSOFT package v4.2 and v6.0.1 (with fastPCA): https://www.hsph.harvard.edu/alkes-price/software/ https://github.com/DReichLab/EIG/<br>SHAPE-IT v2.5: https://mathgen.stats.ox.ac.uk/genetics_software/shapeit/shapeit.html<br>IMPUTE2 v2.3.1: https://mathgen.stats.ox.ac.uk/impute/impute_v2.html

For manuscripts utilizing custom algorithms or software that are central to the research but not yet described in published literature, software must be made available to editors/reviewers. We strongly encourage code deposition in a community repository (e.g. GitHub). See the Nature Research guidelines for submitting code & software for further information.

Data

Policy information about availability of data

All manuscripts must include a data availability statement. This statement should provide the following information, where applicable:

- Accession codes, unique identifiers, or web links for publicly available datasets
- A list of figures that have associated raw data
- A description of any restrictions on data availability

Our meta-analysis summary statistics are publicly available via direct request and at https://github.com/Wittelab/pancancer_pleiotropy. The UKB cohort data is publicly available from the UKB access portal at https://www.ukbiobank.ac.uk. The UKB cancer phenotyping we performed to define cases and associated data dictionary have been provided to the UKB for public use (Return IDs 181 and 183). The Kaiser Permanente data are available via application with a local collaborator at: https://researchbank.kaiserpermanente.org/our-research/for-researchers/.

Field-specific reporting

Please select the one below that is the best fit for your research. If you are not sure, read the appropriate sections before making your selection.

☒ Life sciences ☐ Behavioural & social sciences ☐ Ecological, evolutionary & environmental sciences

For a reference copy of the document with all sections, see nature.com/documents/nr-reporting-summary-flat.pdf

Life sciences study design

All studies must disclose on these points even when the disclosure is negative.

Sample size	Research sample came from two previously existing datasets: the UK Biobank cohort (UKB) and the Kaiser Permanente Genetic Epidemiology Research on Adult Health and Aging cohort (GERA). The UKB is a population-based prospective cohort of 502,611 individuals in the United Kingdom. Study participants were aged 40 to 69 at recruitment between 2006 and 2010, at which time all participants provided detailed information about lifestyle and health-related factors and provided biological samples. GERA participants were drawn from adult Kaiser Permanente Northern California (KPNC) health plan members who provided a saliva sample for the Research Program on Genes, Environment and Health (RPGEH) between 2008 and 2011. Individuals included in this study were selected from the 102,979 RPGEH participants who were successfully genotyped as part of GERA and answered a survey concerning lifestyle and medical history
Data exclusions	Analyses were limited to self-reported European ancestry individuals for whom self-reported and genetic sex matched. To further minimize potential population stratification, we excluded individuals for whom either of the first two ancestry PCs fell outside five standard deviations of the mean of the population. Based on a subset of genotyped autosomal variants with minor allele frequency (MAF) ≥0.01 and genotype call rate ≥97%, we excluded samples with call rates <97% and/or heterozygosity more than five standard deviations from the mean of the population. With the same subset of SNPs, we used KING to estimate relatedness among the samples. We excluded one individual from each pair of first-degree relatives, first prioritizing on maximizing the number of the cancer cases relevant to these analyses and then maximizing the total number of individuals in the analyses. Controls were restricted to individuals who had no record of any cancer in the relevant registries, who did not self-report a prior history of cancer (other than non-melanoma skin cancer), and, if deceased, who did not have cancer listed as a cause of death. For analyses of sex-specific cancer sites (breast, cervix, endometrium, ovary, prostate, and testis), controls were restricted to individuals of the appropriate sex. Our study population ultimately included 413,870 UKB participants and 66,526 GERA participants.
Replication	In lieu of traditional replication, for each of our 18 cancers of interest, we meta-analyzed genome-wide association study summary statistics from two independent samples. We then compared our results with an abstracted list of all genome-wide significant SNPs from relevant GWAS published through June 2018, and we detected 308 independent signals with P<1e-6 that confirmed risk SNPs identified by previous GWAS with P<5e-8. Additionally, for cancers with publicly available summary statistics, we tested our potentially novel SNPs with P<1e-6 for replication, and SNPs that did not replicate were not considered novel.
Randomization	All models were adjusted for age at specimen collection, sex (non-sex-specific cancers only), first ten ancestry PCs, genotyping array (UKB only), and reagent kit used for genotyping (Axiom v1 or v2; GERA only).
Blinding	As both UKB and GERA are population-based prospective cohort studies, there was no intervention with designated treatment and control groups. Additionally, all data were de-identified after sample collection before we received it for analysis. Therefore, blinding was not relevant to our study.

Reporting for specific materials, systems and methods

We require information from authors about some types of materials, experimental systems and methods used in many studies. Here, indicate whether each material, system or method listed is relevant to your study. If you are not sure if a list item applies to your research, read the appropriate section before selecting a response.

Materials & experimental systems

n/a	Involved in the study
☒	☐ Antibodies
☒	☐ Eukaryotic cell lines
☒	☐ Palaeontology
☒	☐ Animals and other organisms
☐	☒ Human research participants
☒	☐ Clinical data

Methods

n/a	Involved in the study
☒	☐ ChIP-seq
☒	☐ Flow cytometry
☒	☐ MRI-based neuroimaging

Human research participants

Policy information about studies involving human research participants

Population characteristics	See above sections on sample size and data exclusions
Recruitment	The UKB is a population-based cohort of 500,000 participants recruited in the United Kingdom between 2006-2010. Approximately 9.2 million individuals aged 40-69 years who lived within 25 miles of one of 22 assessment centers in England, Wales, and Scotland were invited to enter to cohort, and 5.5% participated in the baseline assessment. The UKB is not representative of the general population on a variety of sociodemographic, physical, lifestyle and health-related characteristics, with evidence of a 'healthy volunteer' selection bias, details of which are published elsewhere (Fry et al, Am J Epidemiol 2017;186:1026-34. PMID 28641372).<br><br>The GERA Cohort was developed from a mailed survey sent to all adult members of the Kaiser Permanente Medical Care Plan, Northern California Region (KPNC) who had been members for two years or more in 2007. The membership of KPNC is representative of the general population in the 14 county area in which facilities are located, although the membership is underrepresented for the extremes of income at both ends of the spectrum (dbGaP phs000674.v2.p2).
Ethics oversight	The study was approved by the University of California and KPNC Institutional Review Boards and the UKB data access committee, and informed consent was obtained from all participants.

Note that full information on the approval of the study protocol must also be provided in the manuscript.
